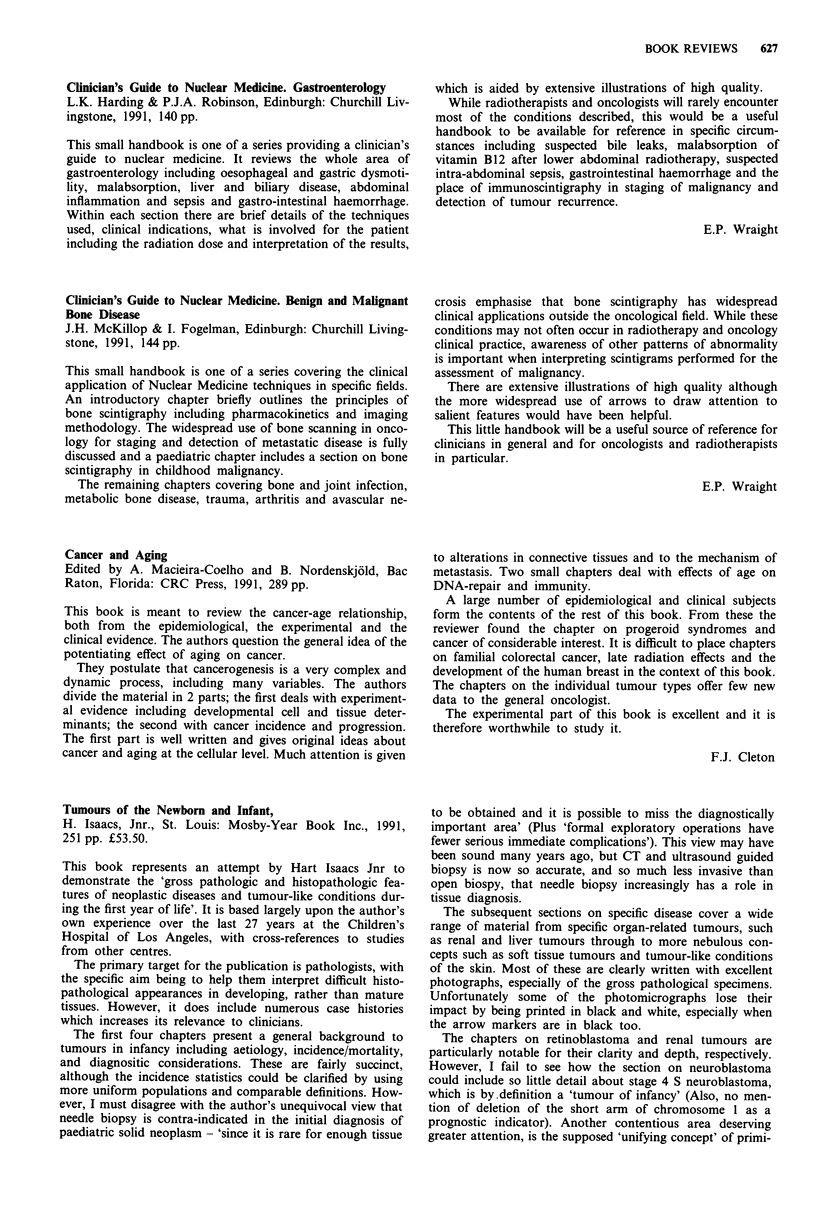# Clinician's Guide to Nuclear Medicine. Benign and Malignant Bone Disease

**Published:** 1992-04

**Authors:** E.P. Wraight


E.P. Wraight

**Clinician's Guide to Nuclear Medicine. Benign and Malignant Bone Disease**
J.H. McKillop & I. Fogelman, Edinburgh: Churchill Livingstone, 1991, 144 pp.

This small handbook is one of a series covering the clinical application of Nuclear Medicine techniques in specific fields. An introductory chapter briefly outlines the principles of bone scintigraphy including pharmacokinetics and imaging methodology. The widespread use of bone scanning in oncology for staging and detection of metastatic disease is fully discussed and a paediatric chapter includes a section on bone scintigraphy in childhood malignancy.

The remaining chapters covering bone and joint infection, metabolic bone disease, trauma, arthritis and avascular necrosis emphasise that bone scintigraphy has widespread clinical applications outside the oncological field. While these conditions may not often occur in radiotherapy and oncology clinical practice, awareness of other patterns of abnormality is important when interpreting scintigrams performed for the assessment of malignancy.

There are extensive illustrations of high quality although the more widespread use of arrows to draw attention to salient features would have been helpful.

This little handbook will be a useful source of reference for clinicians in general and for oncologists and radiotherapists in particular.